# Dataset for the role of sustained attention in memory formation of transgenic mice for Alzheimer׳s disease

**DOI:** 10.1016/j.dib.2016.01.015

**Published:** 2016-01-16

**Authors:** Natalia Mendes Schöwe, Eduardo Moreira de Oliveira, Hudson Sousa Buck, Tania Araujo Viel

**Affiliations:** a Graduate Course on Pharmacology, Institute of Biomedical Sciences, University of Sao Paulo, Sao Paulo, Brazil; b Graduate Course on Neurology, Department of Cognitive Neurology, School of Medicine, University of Sao Paulo, Brazil; c Department of Physiological Sciences, Santa Casa de Sao Paulo School of Medical Sciences, Sao Paulo, Brazil; d School of Arts, Sciences and Humanities, University of Sao Paulo, Sao Paulo, Brazil

**Keywords:** Sustained attention, Memory, Alzheimer׳s disease, Nicotinic, Receptor, Hippocampus

## Abstract

Weekly submission of rats to active avoidance apparatus can be considered a neurostimulation strategy, once it can improve memory and can increase the density of receptors from different neurotransmitter systems in brain areas related to memory. These benefits were observed in rats chronically infused with amyloid-β peptide. In the present work it is presented that the same benefit for memory was observed in five months old transgenic mice for Alzheimer’s disease (TG-PDGFB-APP_Sw,Ind_). However, at this age, no change in density of nicotinic receptors was observed.

**Specifications Table**

**Table:** 

Subject area	Biology
More specific subject area	Neuroscience
Type of data	Graphs, figures
How data was acquired	Behavioural data: Two-way shuttle-box (Ugo Basile, Comerio, Italy); quantification of receptor binding sites: MCID image analysis system (Interfoccus Europe, UK).
Data format	Analyzed
Experimental factors	Mice chronically infused with amyloid-β peptide and submitted weekly to 2-way shuttle-box
Experimental features	Behavioural data was acquired weekly in a two-way shuttle-box. The density of α7 nicotinic receptors was analyzed using autoradiography with [^125^I]-α-bungarotoxin (5 nM). The autoradiography films were analyzed using MCID digital analysis system.
Data source location	Sao Paulo, Brazil
Data accessibility	Data is with this article.

**Value of the data**

• Attention training in a two-way shuttle box can modulate the central nervous system and improve memory.
• Transgenic mice for Alzheimer’s disease, that normally present neuronal loss, also had their memory improved when submitted to this strategy.
• Attention training can be used to stimulate the central nervous system and improve the behavior of other animal models of central diseases.
• At five months of age, these animals did not present a change in the density of α7 nicotinic receptors, there are known to be involved in neuroprotection.

## 1 Data

Rats and mice infused with amyloid-β (Aβ) peptide presented loss in memory due to neurodegeneration of hippocampal and cortical cells [1], [2], [3]. When the animals were weekly submitted to a two-way shuttle box (active avoidance apparatus), their memory was maintained even in the presence of the Aβ [2], [3]. This strategy also increased the density of α7 nicotinic receptors in brain areas related to memory, like cortex, hippocampus and amygdala [3]. The participation of the α7 nicotinic receptors in the maintenance of memory by the submission of animals to the active avoidance apparatus was confirmed in C57Bl/6 mice submitted to the same protocol and also infused with the α7 nicotinic receptors antagonist methyllycaconitine (paper in preparation). In the present work, the same protocol was applied to five months old transgenic mice for Alzheimer׳s disease and the same improvement in behavior was observed. However, no increase in the density of α7 nicotinic receptors was observed in this animal model at this age.

### 1.1 Behavioral data

**Fig. 1:**
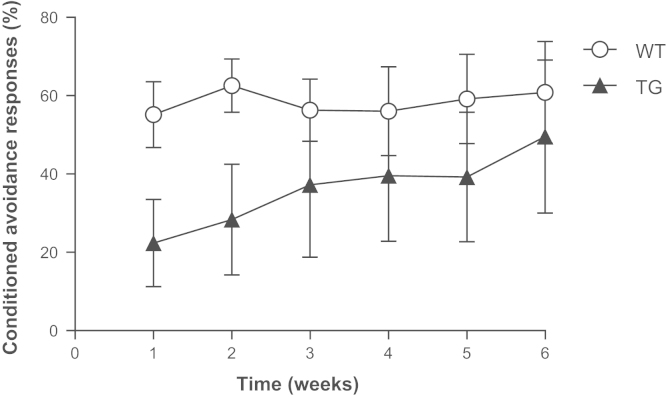
Conditioned avoidance responses of transgenic mice during the six weeks stimulation in active avoidance shuttle-box. Data are means±SEM.

Animals were weekly stimulated in the active avoidance (two-way) shuttle-box during six weeks. In the first two weeks, transgenic mice showed 40.6% and 45.4%, respectively, less conditioned avoidance responses (CAR) than WT control animals (Fig. 1). Along the stimulation process, the animals showed an increase in CAR percentage, getting similar to the WT behavior.

### 1.2 Quantification of α7 nicotinic receptors

**Fig.2:**
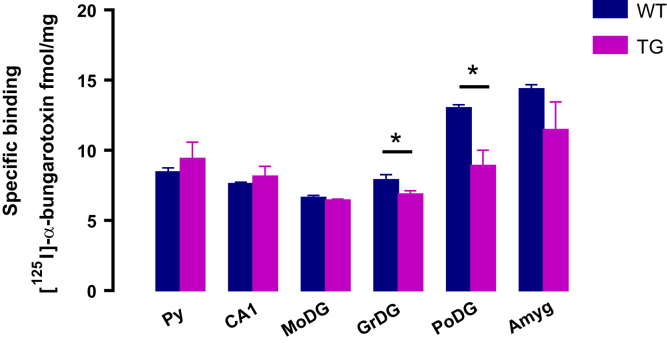
Specific binding of [^125^I]-α-BUTX to α7 nicotinic cholinergic receptor in the hippocampal areas and in the amygdala (Amyg) of the different animal groups. Data are presented as means±SEM.* *P*<0.05.

**Fig. 3:**
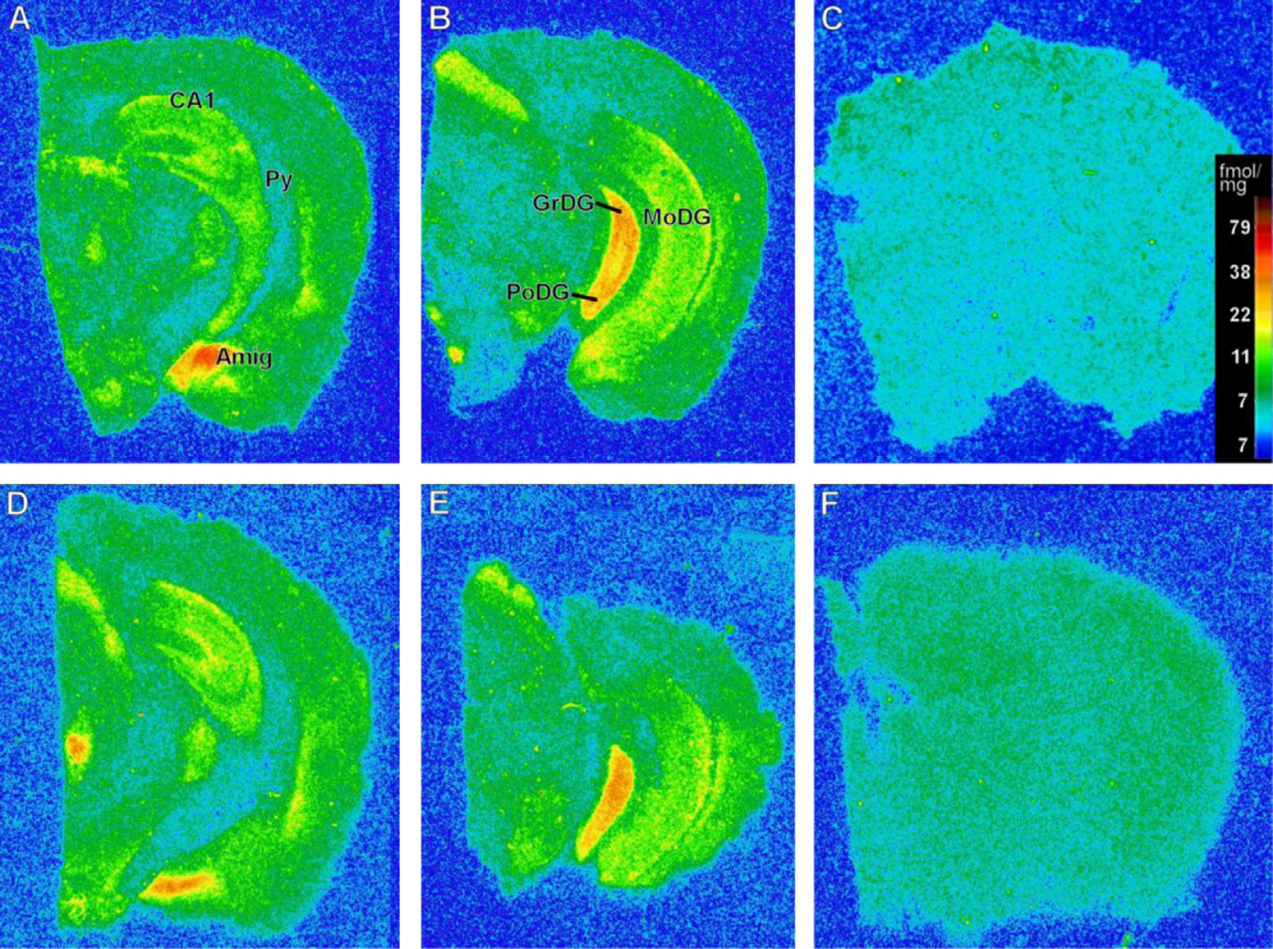
Pseudocolor photomicrographs of autoradiograms representing anatomical distribution of total binding sites for α7 nACh receptor in the pyramidal cells, CA1, molecular (MoDG), granular (GrDG) and plymorphic (PoDG) layers of dentate gyrus and amygdala (amyg) of C57Bl/6 (A, B) and transgenic (D, E) mice. Non-specific binding sites are represented in panels C and F.

The presence of α7 nicotinic cholinergic receptors was verified in many brain areas. In the pyramidal cells of hippocampus (Py), in the CA1 and in the molecular layer of the dentate gyrus (MoDG) no difference between WT and TG animals was verified. The same was verified in the amygdala. However, a significant reduction in α7 density in TG animals was verified in the granular layer of the dentate gyrus (GrDG, 12.6%) and in the polymorphic layer of the dentate gyrus (PoDG, 31.7%) of TG animals, when compared to WT animals (7.90±0.36 fmols/mg and 13.03±8.92 fmols/mg, respectively, Fig.2, Fig. 3).

## 2 Experimental design, materials and methods

### 2.1 Animals

Male hemizygous transgenic mice, with five months of age-here called TG-B6.Cg-Tg (PDGFB-APPSwInd)-that express a mutant form of the human amyloid precursor protein (hAPP) carrying the Swedish (K670N, M671L) and Indiana (V717F) familial Alzheimer׳s disease mutations directed by the platelet-derived growth factor (PDGF) β-chain promoter (APP mice, J20 line) were used as the animal model of neurodegeneration. These mice present loss of synaptic terminals and neuronal bodies at four months of age. Also, at this age they present loss in neuronal function with no senile plaques deposition. The plaques deposition can be observed when animals are 8–10 months of age [4], [5]. Age-matched wild type litter mates-here called WT-were provided from our own colony, using breeding males acquired from “The Jackson Laboratories”, USA (Stock Number 006293). Mice were kept in controlled room temperature (22–24 °C) and humidity (55–65%), with food and water *ad libitum* in a 12-h-light/dark cycle. All the surgery and care procedures were strictly performed according to the guidelines for animal experimentation as stipulated in the Guide for the Care and Use of Laboratory Animals (National Institute of Health Publication number 86-23, Bethesda, MD) and The Ethics Committee on Experimental Research from Faculdade de Ciencias Medicas da Santa Casa de Sao Paulo, Brazil. All efforts were made to minimize the number of animals used and their level of suffering.

### 2.2 Behavioral tests

The active avoidance apparatus was used to evaluate memory evocation and also to stimulate the alert and sustained attention of the animals. Animals were selected according to their ability to learn and memorize a task [1]. For this purpose, a two-way shuttle-box (Ugo Basile, Comerio, Italy) consisting of two compartments accessible to each other by a hole in the wall was used. Each animal was placed individually and then acclimated to the shuttle-box apparatus for 5 min before each session. The animal was then subjected to 50 trials of avoidance conditioning (acquisition test). Each trial consisted of 6 s conditioned stimulus (CS), i.e., a buzzer (70 dB, 760 Hz) and light, followed by a 4 s unconditioned stimulus (UCS), a scramble shock of 0.5 mA delivered through floor grid. Each trial was separated by fixed intertrial intervals (20 s). During the acquisition session the number of conditioned avoidance responses (CAR), in which the animals moved to the other compartment of the shuttle-box before the beginning of the UCS, was recorded. All tests were carried out during the light phase (9:00–16:00 h). All animals were submitted weekly to the same experimental protocol during six consecutive weeks.

### 2.3 Autoradiography for α7 nAChR

After the behavioral protocols, animals were anesthetized and killed by decapitation. The brains were removed and immediately frozen in dimethylbutane and maintained at -80oC until the use.

Serial sections of brains (20 μm) were cut on a cryostat chamber (−20 °C to −22 °C, Microm HM 505 N, Francheville, France), thaw-mounted on gelatin-coated slides, desiccated for 5 min at room temperature and kept at-80 °C until use. Sections were brought to room temperature (22 °C) and air dried (5–10 min). Incubations were conducted for 90 min at room temperature using 5 nM [^125^I]-α-bungarotoxin ([^125^I]-BUTX, 143.2 Ci/mmol). This concentration of radioligand was based on previous studies in the rat brain (data not shown) and corresponds to the Bmax value. Specific binding of the toxin for α7 nAChR, in this concentration, accounted for 80.2% of the total binding. Non-specific binding was assessed using 2 µM of the unlabeled toxin. The radioligand was diluted in 50 mM phosphate buffer containing 1 mM ethilenediethil-tetra-acetic acid (EDTA) and 1 mM phenyl-methil-sulphonyl fluoride (PMSF), pH 7.4. At the end of the incubation period, slides were sequentially transferred through four rinses of 4 min each in the same buffer at 4 °C and rapidly dipped into cold distilled water to remove excess salts. Sections were air-dried and juxtaposed against Hyperfilm-MP (double-coated, 24×30 cm^2^, Amersham Biosciences GE Healthcare, Uppsala, Sweden) for 14 days (room temperature), along with autoradiographic [^125^I] micro scales (20 µm, Amersham Biosciences GE Healthcare). The films were developed in D-19 Kodak developer and fixed in Kodak Ektaflo solution.

### 2.4 Quantification of receptor binding sites

The autoradiograms were quantified densitometrically using the MCID image analysis system (Interfoccus Europe, UK). For each specimen, α7 receptors binding sites were measured on 6-12 sections. The specific binding was determined by subtracting the non-specific binding from the total binding of adjacent sections.

### 2.5 Statistical analysis

Data were expressed as means±S.E.M. Behavioral data was analyzed using two-way repeated measusers ANOVA followed by Bonferroni’s multiple comparisons test. Autoradiography data was analyzed by unpaired Student׳s-*t* test between WT and TG data of each brain area. Only probability values (*P*) less than 0.05 were considered statistically significant.
